# Pulmonary wedge resection for lung cancer developing in a single transplanted lung: a case report

**DOI:** 10.1186/s44215-023-00120-8

**Published:** 2024-01-29

**Authors:** Koji Aze, Masaaki Sato, Masaaki Nagano, Jun Nakajima

**Affiliations:** Department of Thoracic Surgery, The University of Tokyo Hospital, 7-3-1 Hongo, Bunkyo-ku, Tokyo, 113-8655 Japan

**Keywords:** Lung transplantation, Extracorporeal membrane oxygenation, Lung cancer

## Abstract

**Background:**

Primary lung cancer arising in a transplanted lung is much rarer than cancer arising in a native lung. We herein describe a case of lung cancer developing in a transplanted lung after single-lung transplantation. Wedge resection was safely and successfully completed using venovenous extracorporeal membrane oxygenation (VV-ECMO).

**Case presentation:**

A 63-year-old man underwent right single-lung transplantation for idiopathic interstitial pneumonia from a donor in his 60s with a 44-pack-year history of smoking. One year 6 months later, computed tomography revealed a 10-mm nodule in the right lower lobe of the transplanted lung. Fluorodeoxyglucose-positron emission tomography showed abnormal accumulation of fluorodeoxyglucose (maximum standardized uptake value, 2.8) in the same area, suggesting lung cancer. However, percutaneous or transbronchial biopsy was technically impossible. Surgical diagnosis and treatment were planned, and VV-ECMO was introduced before wedge resection. Although pleural adhesion and the location of the nodule close to the hilum resulted in a challenging procedure, the operation was completed in 190 min. The final pathological diagnosis was papillary adenocarcinoma.

**Conclusions:**

Lung cancer arising in a lung transplanted from a brain-dead donor is rare. Limited lung resection using VV-ECMO is considered a useful option, particularly in cases of single-lung transplantation.

## Background

Primary lung cancer in the native lung is a common complication encountered during follow-up of single-lung transplant recipients, with incidence rates ranging from 0.4 to 8.9% [1].

Major indications for lung transplantation are chronic obstructive pulmonary disease (COPD) and interstitial lung disease (ILD). These diseases also serve as independent risk factors for lung cancer. The effects of aging and the recipient’s medical history of exposure to carcinogenic agents (e.g., cigarette smoke) prior to the transplantation procedure may also contribute to the development of lung cancer in the native lung following single-lung transplantation. Among transplant recipients, those who undergo single-lung transplantation are at higher risk for lung cancer because the native lung is left behind after the procedure, thereby remaining exposed to these conditions [2]. By contrast, lung cancer developing in a transplanted lung is much rarer than cancer arising in a native lung, with the prevalence of bronchogenic carcinoma in the transplanted lung ranging from 0.3 to 0.4% [3]. This is because donor lungs with COPD or ILD, which carry a higher risk of carcinogenesis, are typically not used for lung transplantation. Nevertheless, the scarcity of organs and the high mortality rate among waitlisted patients have necessitated the utilization of organs from extended-criteria donors [3, 4]. These donors may be elderly or smokers, which could theoretically increase the likelihood of developing lung cancer.

We herein present a case report of lung cancer originating in a transplanted lung that was successfully treated through surgical intervention using venovenous extracorporeal membrane oxygenation (VV-ECMO).

## Case presentation

A 63-year-old man underwent a single right lung transplantation because of idiopathic interstitial pneumonia. The donor was also a man in his 60s, categorized as a marginal case with a 44-pack-year history of smoking. Because of the recipient’s progressive disease before transplantation, the marginal donor was accepted. The transplantation was uneventful, although home oxygen therapy during exertion was required after the transplantation.

**Fig. 1:**
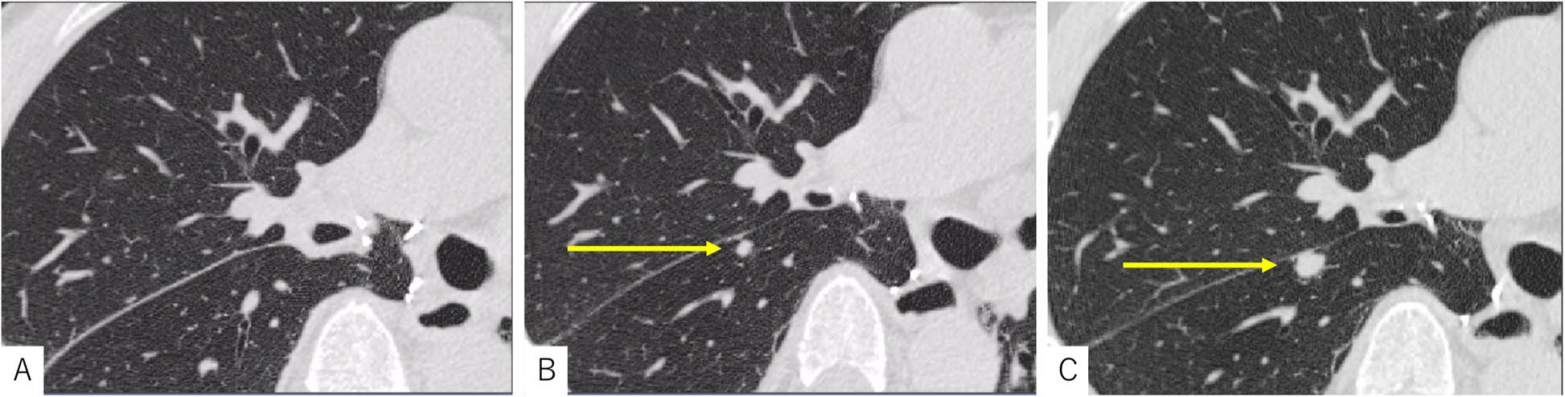
Changes in the pulmonary nodule on chest computed tomography. **A** Three months after right single-lung transplantation, the nodule was hardly identifiable. **B** The nodule was first recognized 1 year after right single-lung transplantation (arrow). **C** At 18 months after lung transplantation, the nodule was enlarged (arrow)

One year after the transplantation, computed tomography (CT) revealed a small nodule in the right lower lobe of the transplanted lung. After 6 months of follow-up, the nodule was found to have enlarged to 10 mm in diameter (Fig. 1). Laboratory studies revealed elevated serum carcinoembryonic antigen (8.9 ng/mL) and cytokeratin 19 fragment levels (3.5 ng/mL). Positron emission tomography-CT showed abnormal accumulation of fluorodeoxyglucose (maximum standardized uptake value, 2.8) in the same area, which suggested a diagnosis of lung cancer (cT1aN0M0, stage IA1). However, percutaneous or transbronchial biopsy was technically impossible. Consequently, surgical diagnosis and treatment were planned.

The nodule had developed in the transplanted lung, and the operation plan involved a reduction of the pulmonary resection volume as much as possible. We opted to perform pulmonary wedge resection. Because single-lung ventilation of the native lung with pulmonary fibrosis was evidently insufficient to maintain the patient’s oxygenation during the procedure, the introduction of VV-ECMO was planned. The internal jugular vein was deemed inappropriate for blood access because of suspected stenosis of the superior vena cava on CT images. We were willing to do a central cannulation from the right thoracotomy to the right atrium if possible.

Under general anesthesia and two-lung ventilation, the patient was positioned in a semi-left lateral decubitus position. We performed an open thoracotomy using the previous skin incision. After dissection of severe pleural adhesion, we reached the pericardium. However, central cannulation did not appear to be easy from the right thoracotomy. Thus, the bilateral femoral veins were cannulated; the inflow cannula was inserted through the right femoral vein to the right atrium and the outflow cannula was inserted through the left femoral vein to the inferior vena cava. VV-ECMO was initiated and one-lung ventilation with the patient’s left native lung was started. The right transplanted lung was deflated. Despite the concern regarding recirculation, oxygenation was successfully maintained during the operation. We continuously infused unfractionated heparin to maintain activated clotting time at approximately 200 s during the use of VV-ECMO.

**Fig. 2:**
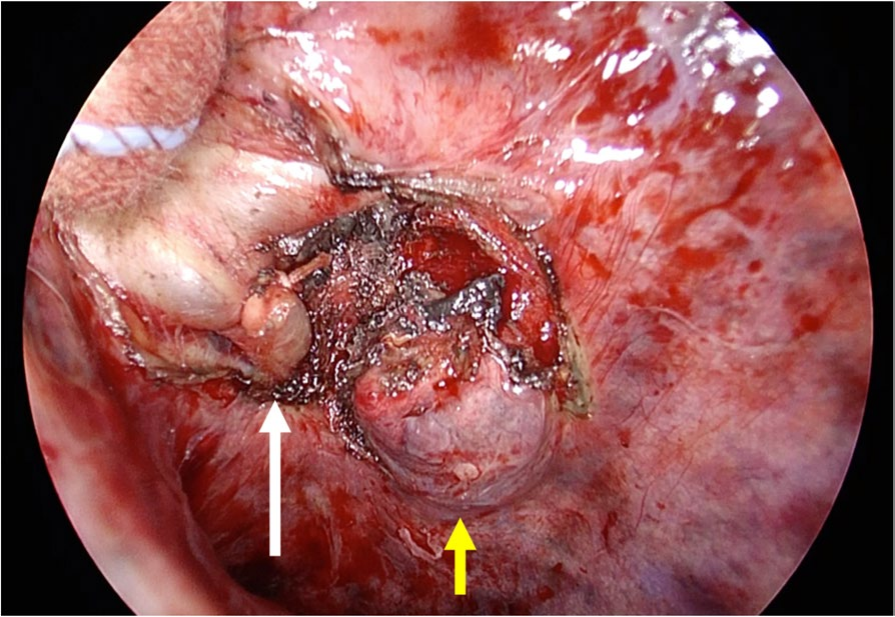
Intraoperative photograph showing the nodule (yellow arrow) located close to the hilar pulmonary artery at the interlobar fissure. A pulmonary arterial branch (A6a) was ligated and transected (white arrow)

The adhesion in the interlobar fissure was relatively loose, allowing blunt dissection. The nodule was located in the superior segment of the lower lobe, close to the hilum but facing the interlobar fissure. The nodule was removed by precision cautery excision (Perelman technique) [5] with some modification. The pulmonary arterial branch (A6a) close to the nodule was ligated and transected, and the nodule was excised with some surrounding lung tissue using electrocautery (Fig. 2). The nodule and the surrounding lung tissue were then lifted and clamped with Kelly forceps, the nodule was resected, and then the remaining lung tissue was manually sutured. No obvious air leakage was observed after resuming bilateral ventilation. The chest was closed, leaving a chest tube behind, and VV-ECMO was withdrawn. The peripheral capillary oxygen saturation was sustained at approximately 90% throughout the procedure, and the operation was completed in 190 min. The estimated intraoperative blood loss was 80 g.

**Fig. 3:**
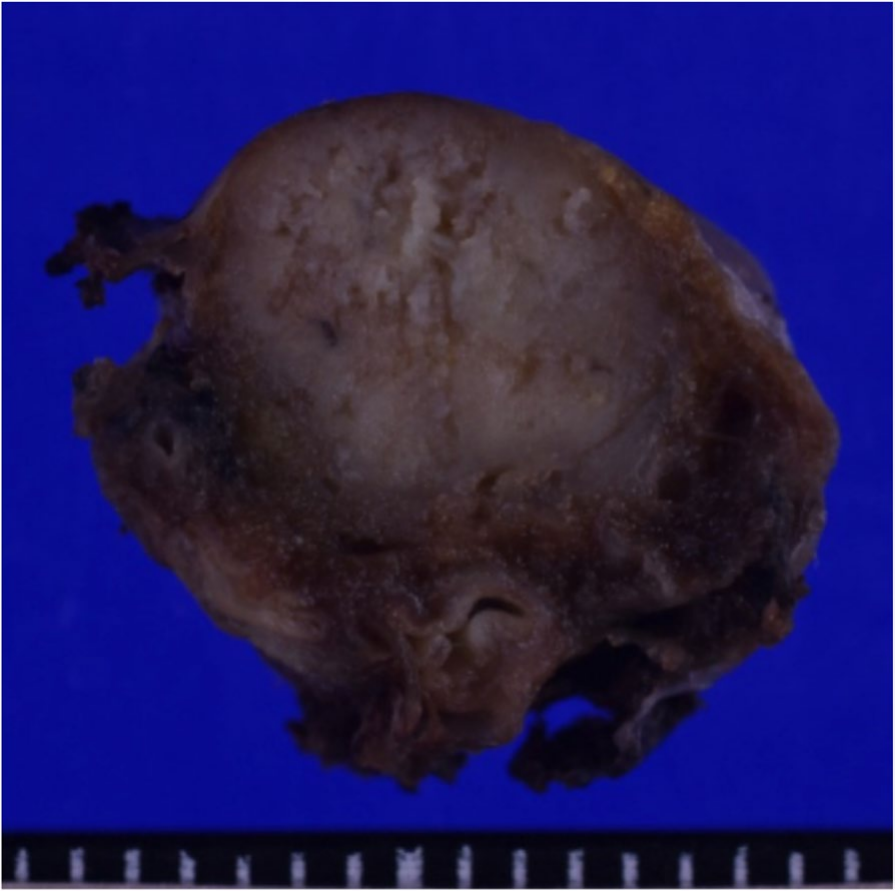
The surgical specimen was 13 × 9 × 8 mm in size, and the surgical margin was close but negative

The final pathologic diagnosis was papillary adenocarcinoma (pT1bN0M0, stageIA2), and the surgical margin was close but negative (Fig. 3). The post-transplant triple immunosuppression regimen was continued after the operation although the target trough level of tacrolimus was reduced from 8 to 10 ng/ml to 5–7 ng/ml and minimal doses of mycophenolate mofetil (250 mg bid) and prednisolone (4 mg/day) were maintained. Postoperative CT showed no remaining lung cancer. The patient had been disease-free for 12 months postoperatively without any additional therapy.

## Discussion and conclusions

Recipients of solid organ transplantation are at significantly higher risk of cancer development than the general population [6]. This is believed to be due to the need for chronic immunosuppressive therapy after transplantation, which impairs anti-tumor immune surveillance and anti-viral activity, thereby playing a central role in cancer development [7, 8]. Lung transplant recipients typically receive higher doses of immunosuppression than other solid organ transplant populations, which likely contributes to the higher rates of cancer observed in this group [9].

Primary lung cancer in the native lung is a common complication encountered during follow-up of single-lung transplant recipients [1], whereas the occurrence of lung cancer in the transplanted lung in general is much less common than in the native lung (0.3 to 0.4%) [3]. However, as was the case in the donor of the present report, the scarcity of organs and the high mortality rate among waitlisted patients have necessitated the utilization of organs from extended-criteria donors including those over the age of 55 years and with a significant history of smoking [3, 4]. With the increasing use of older donors and their extensive smoking history, the risk of transplanting cancer cells from the donor to the recipient may increase in the future [10–12]. When the donor’s lung exhibits triple criteria such as heavy smoking history, high age, and cystic or fibrotic changes, careful selection of the recipient is imperative. However, if a potential recipient needs relatively urgent transplantation, we consider such a donor lung acceptable for transplantation as long as it is functionally satisfactory. Nonetheless, frequent follow-up on the transplanted lung after transplantation is required.

Treatment of newly diagnosed lung cancer in transplant recipients should follow the current guidelines, although some treatment options may be limited by the patient’s health condition and post-transplant morbidity [13].

In our patient, wedge resection was planned to minimize the amount of lung tissue resected and thus preserve the respiratory function after surgery. ECMO was introduced before wedge resection to maintain oxygenation during the operation. In selecting blood access, venoarterial ECMO was excluded because of its expected association with central hypoxia. VV-ECMO was chosen, but careful consideration was still required with this blood access. We finally applied a femorofemoral circuit configuration. Monitoring of pulmonary artery pressure was not performed during the surgery because there were no findings on echocardiography suggesting right heart failure or elevated pulmonary artery pressure.

Although ECMO was not required during the right single-lung transplantation, it was necessary for lung cancer surgery in the transplanted lung. After single-lung transplantation, most lung perfusion is shifted toward the transplanted lung [14]. If one-lung ventilation with the native lung is performed, arterial blood oxygen saturation will decrease because of intrapulmonary shunting in the transplanted lung.

In this case, after the right single-lung transplantation, the recipient’s total perfusion was distributed 72.5% to the right side (transplanted side) and 27.5% to the left side (native lung), in contrast to the pre-transplantation distribution of 45% to the right side and 55% to the left side. In addition, the progression of pulmonary fibrosis over time made it difficult to maintain oxygenation with one-lung ventilation by the native lung. Thus, ECMO induction was a useful option in this case to ensure successful lung cancer surgery.

In conclusion, we successfully conducted surgical resection of lung cancer arising in the lung transplanted from a brain-dead donor after single-lung transplantation. VV-ECMO was successfully used to overcome the challenge of single-lung ventilation of the remaining native lung during the procedure. Primary lung cancer developing in a transplanted lung is a potential concern when extended-criteria donors of advanced age and/or with a significant smoking history are involved. Close attention should be paid during follow-up in such cases.

## Data Availability

Not applicable.

## Abbreviations

COPD: Chronic obstructive pulmonary disease
ILD: Interstitial lung disease
CT: Computed tomography
VV-ECMO: Venovenous extracorporeal membrane oxygenation
